# Three new species of the segmented spider genus *Qiongthela* (Mesothelae, Liphistiidae) from Hainan Island, China

**DOI:** 10.3897/zookeys.1009.57857

**Published:** 2021-01-11

**Authors:** Li Yu, Fengxiang Liu, Zengtao Zhang, Daiqin Li, Xin Xu

**Affiliations:** 1 College of Life Sciences, Hunan Normal University, Changsha, Hunan 410081, China Hunan Normal University Changsha China; 2 State Key Laboratory of Biocatalysis and Enzyme Engineering, and Centre for Behavioural Ecology and Evolution (CBEE), School of Life Sciences, Hubei University, 368 Youyi Road, Wuhan, Hubei 430062, China Hubei University Hubei China; 3 Department of Biological Sciences, National University of Singapore, 14 Science Drive 4, Singapore 117543, Singapore National University of Singapore Singapore Singapore

**Keywords:** DNA barcode, morphology, taxonomy, trapdoor spiders

## Abstract

We report three new species of the segmented trapdoor spider genus *Qiongthela* Xu & Kuntner, 2015 collected from Hainan Island, China based on morphological characters: *Q. dongfang***sp. nov.** (♂♀), *Q. nankai***sp. nov.** (♂♀), *Q. yalin***sp. nov.** (♂♀). We also provide the GenBank accession codes of the DNA barcode gene, cytochrome c oxidase subunit I (COI), of the type specimens of all three new species to aid future identification.

## Introduction

**Figure 1.**
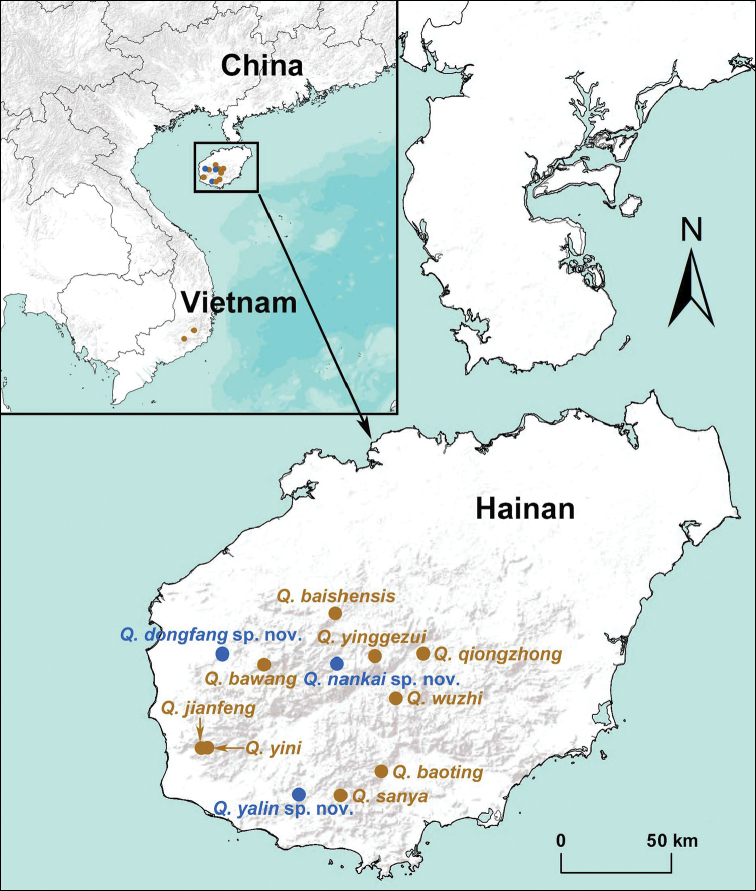
Map showing the type localities of fourteen *Qiongthela* species in southern Vietnam and Hainan Island, China. The type localities of 11 known species are indicated in brown solid circles, and the three new species are indicated in blue solid circles.

The segmented trapdoor spider genus *Qiongthela* Xu & Kuntner, 2015 is currently distributed in Hainan Island (China) and southern Vietnam (Xu et al. 2015a, b; World Spider Catalog 2020; Fig. 1). It contains 11 described species, nine of which have been recorded from Hainan Island and the remaining two, *Q. australis* (Ono, 2002) and *Q. nui* (Schwendinger & Ono, 2011), found in southern Vietnam (Ono 2002; Schwendinger and Ono 2011; Xu et al. 2015b; Yu et al. 2020; Word Spider Catalog 2020).

When examining the specimens collected from Hainan Island, we diagnosed three new *Qiongthela* species. Here, we describe these new species based on genital morphology of both males and females. Furthermore, we provide the genetic distances of intraspecific and interspecific relationships with the closest species based on the DNA barcode gene, cytochrome c oxidase subunit I (COI), to support our descriptions, and also provide the COI sequences of type specimens for future identifications.

## Materials and methods

All specimens in this study were collected from Hainan Island, China. We collected them alive, checked for their maturity status, removed the right four legs of adult specimens, preserved the legs in 100% ethanol, and kept them at –80 °C for molecular work. The remains were preserved in 80% ethanol as vouchers for morphological identification and examination. We took juvenile/subadult males back to the laboratory, reared them until they reached sexual maturity, removed the right four legs, and preserved them as described above. All the types and voucher specimens are deposited at the College of Life Sciences, Hunan Normal University, Changsha, Hunan Province, China.

We examined and dissected the specimens using an Olympus SZX16 stereomicroscope. We removed the soft tissues of female genitalia using 10 mg/ml trypsase (Bomei Biotech Company, Hefei, Anhui, China) for at least 3 hours at room temperature. We took photographs of male and female genitals under an Olympus BX53 compound microscope using a CCD digital camera. We conducted all measurements using an MC170HD digital camera mounted on a Leica M205C stereomicroscope and presented the measurements in millimeters. Leg and palp measurements are given in the following order: leg total length (femur + patella + tibia + metatarsus + tarsus), palp total length (femur + patella + tibia + tarsus).

Abbreviations used: ALE = anterior lateral eyes; AME = anterior median eyes; BL = body length; CL = carapace length; Co = conductor; CT = contrategulum; CW = carapace width; E = embolus; HNU = Hunan Normal University; OL = opisthosoma length; OW = opisthosoma width; PC = paracymbium; PLE = posterior lateral eyes; PME = posterior median eyes; RC = receptacular cluster; T = tegulum.

We extracted total genomic DNA from spider legs using the Animal Genomic DNA Isolation Kit (Kangwei Biotech, Beijing, China) following the manufacturer’s protocols. We used the primer pair LCO1490/HCO2198 (Folmer et al. 1994) to amplify COI sequences under the following PCR reaction protocol: initial denaturation at 95 °C for 5 min; 35 cycles of denaturation at 95 °C for 1 min, annealing at 40 °C for 1 min, and elongation at 72 °C for 30 s; and final extension at 72 °C for 7 min (Xu et al. 2015c). The 25 μl PCR reactions consisted of 12.5 μl of 2×Taq MasterMix (KangWei Biotech, Beijing, China), 1 μl of each forward and reverse 10 μM primer, 1 μl of genomic DNA, and 9.5 μl of double-distilled H_2_O. The PCR products were examined using agarose gel electrophoresis (1% agarose). All PCR products were purified and sequenced at Tsingke Biotechnology Company (Changsha, China). We downloaded all the COI sequences of known *Qiongthela* species from NCBI and calculated genetic distances based on the standard DNA barcode alignment using MEGA v6.0 (Tamura et al. 2013).

## Taxonomy

### Genus Qiongthela

Taxon classificationAnimaliaAraneaeLiphistiidae

Xu & Kuntner, 2015

72246532-551C-5AB1-B92F-85C1CD82344A

#### Type species.

*Qiongthela baishensis* Xu, 2015.

#### Diagnosis.

Males of *Qiongthela* can be distinguished from those of all other six Heptathelinae genera by the blade-like conductor narrowing towards the apex (Figs 3A–C, E, 5A–E, 7A–E), and by the tegulum with two obvious apophyses (Figs 3A–E, 5A–E, 7A–E). Females of *Qiongthela* differ from those of all other six Heptathelinae genera by the two pairs of the receptacular clusters with numerous granula (Figs 3H–K, 5H–K, 7H–M) (Xu et al. 2017; Yu et al. 2020).

#### Species composition.

*Q. australis* (Ono, 2002), *Q. baishensis* Xu, 2015, *Q. baoting* Yu, Liu, Zhang, Wang, Li & Xu, 2020, *Q. bawang* Xu, Liu, Kuntner & Li, 2017, *Q. jianfeng* Xu, Liu, Kuntner & Li, 2017, *Q. nui* (Schwendinger & Ono, 2011), *Q. qiongzhong* Yu, Liu, Zhang, Wang, Li & Xu, 2020, *Q. sanya* Yu, Liu, Zhang, Wang, Li & Xu, 2020, *Q. wuzhi* Xu, Liu, Kuntner & Li, 2017, *Q. yinggezui* Yu, Liu, Zhang, Wang, Li & Xu, 2020, *Q. yini* Xu, Liu, Kuntner & Li, 2017.

#### Distribution.

China (Hainan), Vietnam.

### Qiongthela dongfang sp. nov.

Taxon classificationAnimaliaAraneaeLiphistiidae

EE418EA5-3BEB-5966-9E06-45CF8DAC4CFE

http://zoobank.org/6A654737-8B5C-48F0-84F6-BC0C14333BAD

Figures 2 , 3

#### Type material.

***Holotype*** ♂: China, Hainan Province, Dongfang City, between Puguang and the 14^th^ Dongfang Farm, 19.08°N, 108.92°E, alt. 160 m, 24 August 2019, D. Li, F.X. Liu, X. Xu and L. Yu leg., XUX–2019–159 (matured on 2 October 2019 at HNU). ***Paratypes***: 3 ♀♀, same data as holotype, XUX–2019–156, 157, 160; 1 ♂, same locality as holotype, 5 August 2017, D. Li, F.X. Liu, Z.T. Zhang and X. Xu leg., XUX–2017–065 (♂ matured on 20 October 2018 at HNU).

#### Diagnosis.

Males of *Q. dongfang* sp. nov. resemble those of *Q. jianfeng*, but can be distinguished from the latter by the tegular marginal apophysis with a pointed, sharp apex (Fig. 3D, E, G) and the tegular terminal apophysis with a hook-like apex (Fig. 3A, B); from those of the rest of *Qiongthela* species by the conductor base with a semioval apophysis ventrally (Fig. 3E). Females of *Q. dongfang* sp. nov. differ from those of *Q. bawang* and *Q. qiongzhong* by the receptacular clusters all similar in size and with short genital stalks (Fig. 3H–K); from those of *Q. nankai* sp. nov. by the bases of middle receptacular clusters separated from each other (Fig. 3H–K); from those of the rest of *Qiongthela* species by the two paired receptacular clusters separated from each other, and situated along the anterior margin of the bursa copulatrix and all similar in size (Fig. 3H–K).

#### Description.

**Figure 2.**
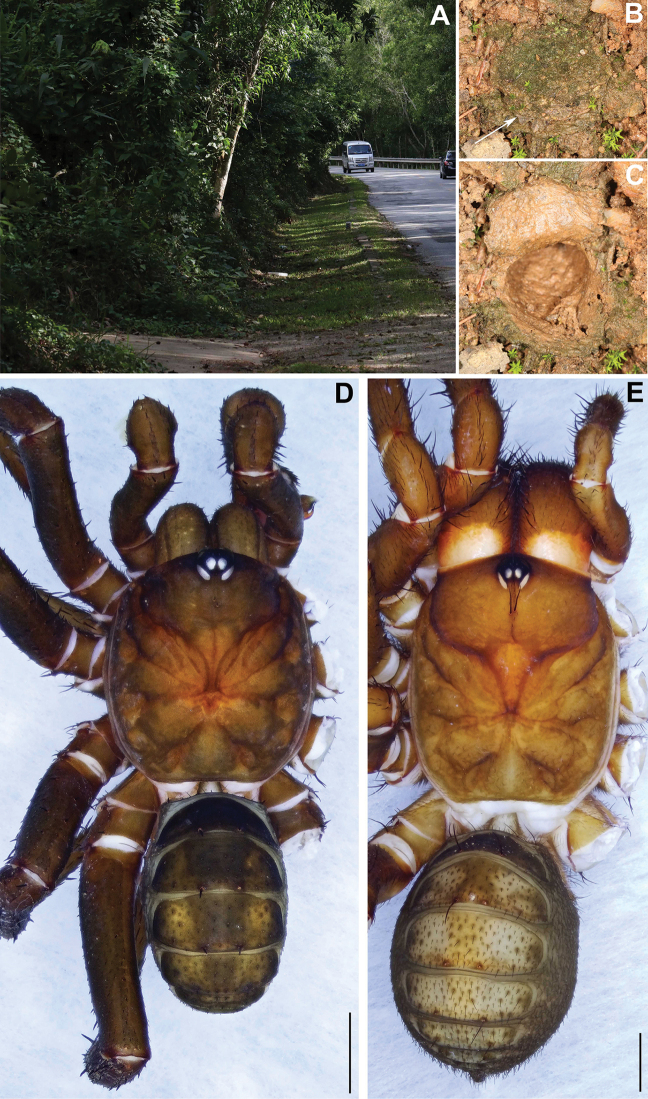
Microhabitat and general somatic morphology of *Qiongthela dongfang* sp. nov. **A** microhabitat **B, C** trapdoor with door closed and open **D** male (XUX–2019–159, holotype) **E** female (XUX–2019–157); Scale bars: 2 mm (**D, E**).

**Male** (holotype, Fig. 2D). Carapace light brown; opisthosoma brown, with 12 dark brown tergites, close to each other, the first 2–7 larger than the others, and the fourth largest; sternum narrow, much longer than wide; a few fine pointed hairs running over the ocular area; chelicerae with promargin of cheliceral groove bearing 8 denticles; legs with firm hairs and spines; 7 spinnerets. Measurements: BL 10.89, CL 5.35, CW 4.85, OL 5.26, OW 3.56; ALE > PLE > PME > AME; leg I 16.22 (4.82 + 1.45 + 3.59 + 4.33 + 2.03), leg II 15.90 (4.48 + 1.30 + 3.58 + 4.37 + 2.17), leg III 15.34 (3.83 + 1.08 + 3.25 + 4.63 + 2.55), leg IV 20.64 (4.95 + 1.10 + 4.43 + 7.00 + 3.16).

**Figure 3.**
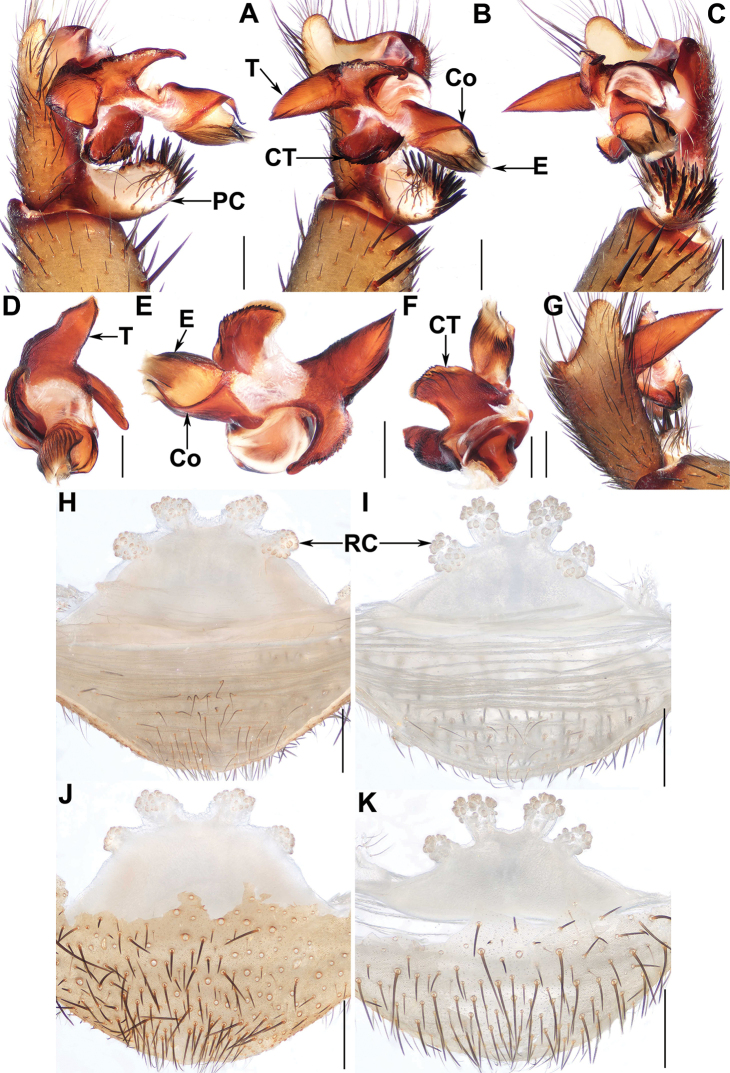
Male and female genital anatomy of *Qiongthela dongfang* sp. nov. **A** left palp, prolateral view **B, E** left palp, ventral view **C** left palp, retrolateral view **D** left palp, distal view **F, G** left palp, dorsal view **H, I** vulva, dorsal view **J, K** vulva, ventral view **A–C, G** XUX–2019–159 (holotype) **D–F** XUX–2017–065 **H, J** XUX–2019–157 **I, K** XUX–2019–160; Scale bars: 0.5 mm.

***Palp*.** Cymbium with a short projection dorsally (Fig. 3G); paracymbium unpigmented and unsclerotised prolaterally, with numerous setae at the tip (Fig. 3A–C). Contrategulum with two edges distally: the inner one finely dentate, the outer one sharp, semi-translucent (Fig. 3D–F). Marginal apophysis of the tegulum long, pointed, wide basally, with a sharp apex (Fig. 3D), proximally-directed terminal apophysis of tegulum with several denticles, narrowing to a hooked apex (Fig. 3A, B, E). Conductor situated ventro-proximally on the embolus, basal portion fused with the embolus and forming a semioval apophysis ventrally, distal portion free, narrowing to a slightly bent apex (Fig. 3A, B, E). Embolus largely sclerotised, with a wide, flat opening of the sperm duct distally (Fig. 3B, C, E).

**Female** (Fig. 2E). Carapace reddish brown; opisthosoma brown, with 12 light brown tergites, close to each other, the first 2–7 larger than the others, and the fourth largest; sternum narrow, much longer than wide; a few fine pointed hairs running over the ocular area; chelicerae robust with promargin of cheliceral groove containing 10 denticles of variable size; legs with firm hairs and spines; 7 spinnerets. Measurements: BL 17.47, CL 8.25, CW 6.78, OL 8.36, OW 6.43; ALE > PLE > PME > AME; palp 11.01 (3.66 + 1.40 + 2.81 + 3.14), leg I 15.16 (4.87 + 1.89 + 3.54 + 3.07 + 1.79), leg II 14.86 (4.78 + 1.85 + 3.20 + 3.15 + 1.88), leg III 15.37 (4.37 + 1.94 + 3.04 + 3.74 + 2.28), leg IV 22.99 (6.70 + 2.58 + 4.55 + 6.06 + 3.10).

***Female genitalia*.** Two pairs of similar-sized receptacular clusters along the anterior margin of the bursa copulatrix, with short genital stalks (Fig. 3H–K).

#### Variation.

Males and females vary in body size. Range of measurements in males (*N* = 2): BL 10.89–14.76, CL 5.35–7.20, CW 4.85–6.50, OL 5.26–7.18, OW 3.56–4.58; in females (*N* = 3): BL 12.63–17.47, CL 6.00–8.25, CW 5.46–6.78, OL 6.19–8.36, OW 4.93–6.43.

#### Etymology.

The species epithet, a noun in apposition, refers to the type locality.

#### Distribution.

Hainan (Dongfang), China

#### GenBank accession number.

XUX–2019–159: MT900751.

#### Remarks.

The maximum and mean intraspecific genetic distances of *Q. dongfang* sp. nov. are 0.3% and 0.2% based on Kimura 2-parameter (K2P) model, respectively (*N* = 5). We calculated the interspecific genetic distance between the holotypes of the two closest species. The genetic distances between *Q. dongfang* sp. nov. and *Q. jianfeng* (GenBank accession code: KP229838 (paratype); we used the sequence of paratype because the DNA barcodes of the holotype and paratype are identical; Xu et al. 2017), *Q. nankai* sp. nov., and *Q. yalin* sp. nov. are 7.7%, 15.7%, and 9.1% based on K2P, respectively.

### Qiongthela nankai sp. nov.

Taxon classificationAnimaliaAraneaeLiphistiidae

66AD90D3-1948-5037-B27E-AEA229378ADC

http://zoobank.org/7A75CB1F-E032-40BC-906D-F79CEAC64B96

Figures 4 , 5

#### Type material.

***Holotype*** ♂: China, Hainan Province, Baisha City, Nankai Town, Nankai Village, 19.04°N, 109.39°E, alt. 300 m, 26 August 2019, D. Li, F.X. Liu, X. Xu and L. Yu leg., XUX–2019–174 (matured on 10 September 2019 at HNU). ***Paratypes***: 1 ♂, 2 ♀♀, same data as holotype, XUX–2019–172, 173, 175.

#### Diagnosis.

Males of *Q. nankai* sp. nov. can be distinguished from those of *Q. qiongzhong*, *Q. yalin* sp. nov. and *Q. yinggezui* by the straight tegular marginal apophysis (Fig. 5A, D), and the cymbial projection short and thick (Fig. 5G); from those of *Q. australis* by the conductor with a bent apex (Fig. 5C, E, G), and the longer tegular marginal apophysis (Fig. 5A, C, D, F); from those of *Q. dongfang* sp. nov., *Q. jianfeng* and *Q. sanya* by the tegular terminal apophysis with an abruptly narrowed and hooked apex (Fig. 5A–E); from those of *Q. nui* by the tegular marginal apophysis with a blunt edge (Fig. 5A–G); from those of the rest of *Qiongthela* species by the contrategulum with two distal edges (Fig. 5A, D). Females of *Q. nankai* sp. nov. differ from those of *Q. dongfang* sp. nov. and *Q. wuzhi* by the middle receptacular clusters situated close to each other (Fig. 5H–K); from those of *Q. bawang* and *Q. qiongzhong* by the two pairs of receptacular clusters similar in size and shape (Fig. 5H–K); from those of the rest of *Qiongthela* species by similar-sized receptacular clusters, and all situated along the anterior margin of the bursa copulatrix (Fig. 5H–K).

#### Description.

**Figure 4.**
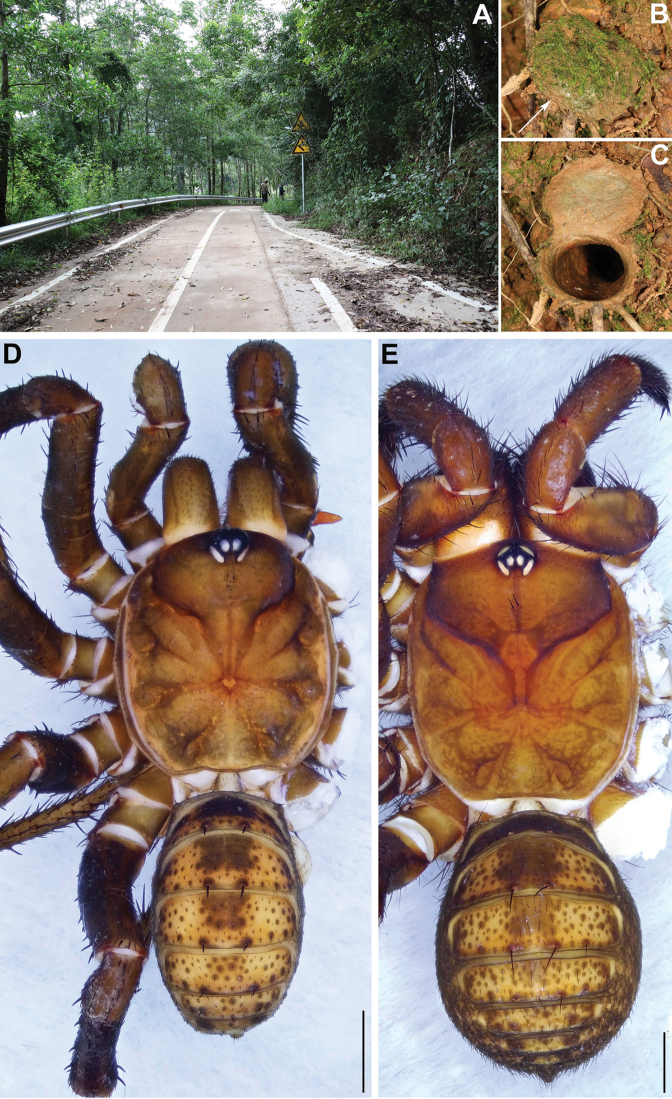
Microhabitat and general somatic morphology of *Qiongthela nankai* sp. nov. **A** microhabitat **B, C** trapdoor with door closed and open **D** male (XUX–2019–174, holotype) **E** female (XUX–2019–173); Scale bars: 2 mm (**D, E**).

**Male** (holotype, Fig. 4D). Carapace reddish brown; opisthosoma brown, with 12 yellow tergites, close to each other, the first 2–7 larger than the others, and the fourth largest; sternum narrow, much longer than wide; a few fine pointed hairs running over the ocular area; chelicerae with promargin of cheliceral groove bearing 11 denticles of variable size; legs with firm hairs and spines; 7 spinnerets. Measurements: BL 11.44, CL 5.31, CW 5.12, OL 5.63, OW 3.62; ALE > PLE > PME > AME; leg I 14.01 (3.91 + 0.95 + 3.18 + 3.94 + 2.03), leg II 14.76 (4.05 + 1.30 + 3.28 + 4.00 + 2.13), leg III 15.64 (3.94 + 1.53 + 3.09 + 4.61 + 2.47), leg IV 21.12 (5.48 + 1.34 + 4.56 + 6.43 + 3.31).

**Figure 5.**
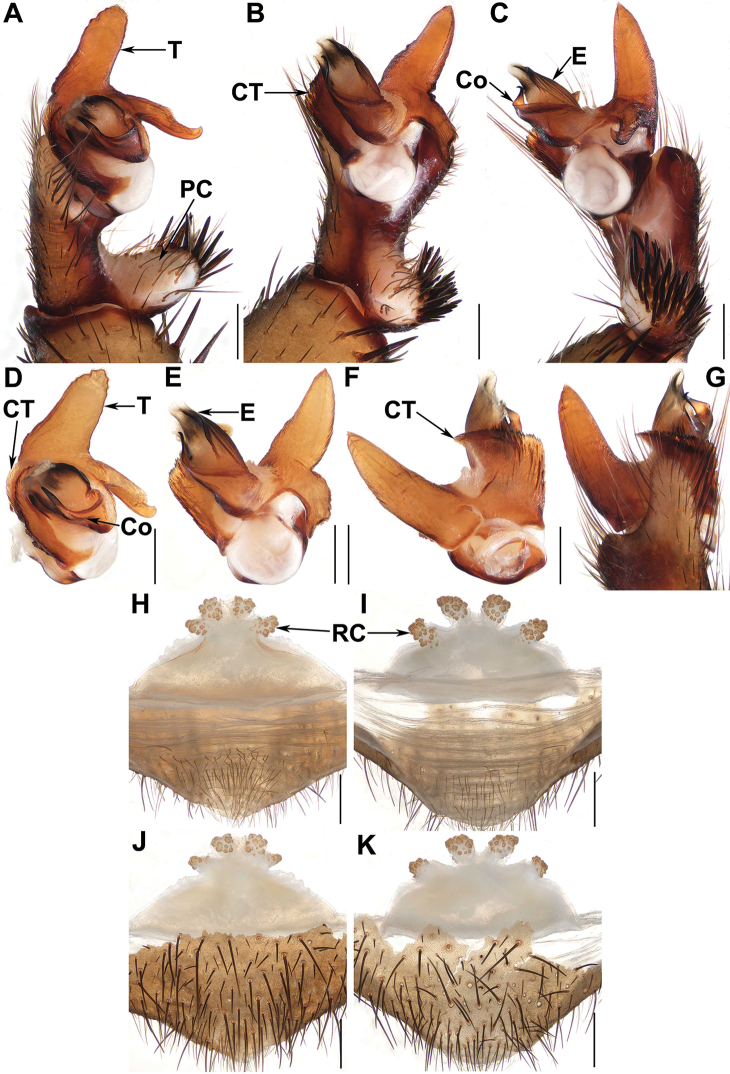
Male and female genital anatomy of *Qiongthela nankai* sp. nov. **A** left palp, prolateral view **B, E** left palp, ventral view **C** left palp, retrolateral view **D** left palp, distal view **F, G** left palp, dorsal view **H, I** vulva, dorsal view **J, K** vulva, ventral view **A–C, G** XUX–2019–174 (holotype) **D–F** XUX–2019–172 **H, J** XUX–2019–173 **I, K** XUX–2019–175; Scale bars: 0.5 mm.

***Palp*.** Cymbium with a short projection dorsally (Fig. 5G); paracymbium unpigmented and unsclerotised prolaterally, with numerous setae at the tip (Fig. 5A–C). Contrategulum with an irregular dentate edge proximally and two edges distally: the inner one dentate, and the outer one sharp, semi-translucent (Fig. 5A, D, F). Marginal apophysis of tegulum long, with a blunt apex distally, distal portion of similar width as basal portion (Fig. 5A, D), a proximally directed terminal apophysis of the tegulum with few denticles, abruptly narrowing to a hooked apex (Fig. 5A–E). Conductor situated ventro-proximally on the embolus, the basal portion fused with the embolus, distal portion free, narrowing to a bent apex (Fig. 5A–E). Embolus largely sclerotised, smooth ventrally, with several longitudinal ribs retrolaterally, and with a wide, flat opening of the sperm duct distally (Fig. 5A, D, E).

**Female** (Fig. 4E). Carapace and opisthosoma color like in male, 12 opisthosoma tergites, close to each other, the first 2–7 larger than the others, and the fourth largest; sternum narrow, much longer than wide; a few fine pointed hairs running over the ocular area; chelicerae robust with promargin of cheliceral groove containing 10 denticles of variable size; legs with firm hairs and spines; 7 spinnerets. Measurements: BL 17.48, CL 8.45, CW 7.56, OL 8.47, OW 6.49; ALE > PLE > PME > AME; palp 14.11 (5.02 + 1.93 + 3.41 + 3.75), leg I 15.49 (4.90 + 1.72 + 3.72+ 3.21 + 1.94), leg II 15.79 (4.91 + 1.97 + 3.41 + 3.38 + 2.12), leg III 16.31 (4.64 + 1.93 + 3.50 + 4.00 + 2.24), leg IV 23.11 (6.47 + 2.18 + 5.08 + 6.21 + 3.17).

***Female genitalia*.** Two pairs of receptacular clusters along the anterior margin of the bursa copulatrix, of similar size and shape, and the middle ones close to each other, with very short genital stalks (Fig. 5H–K).

#### Variation.

Males and females vary in body size. Range of measurements in males (*N* = 2): BL 11.44–12.13, CL 5.31–5.65, CW 5.12–5.56, OL 5.63–6.35, OW 3.62–4.69; in females (*N* = 2): BL 13.09–17.48, CL 6.63–8.45, CW 6.04–7.56, OL 6.32–8.47, OW 5.05–6.49.

#### Etymology.

The species epithet, a noun in apposition, refers to the type locality.

#### Distribution.

Hainan (Baisha), China

#### GenBank accession number.

XUX–2019–174: MT900752.

#### Remarks.

The maximum and mean intraspecific genetic distances of *Q. nankai* sp. nov. are 0.6% and 0.3% based on K2P, respectively (*N* = 4). The interspecific genetic distances between *Q. nankai* sp. nov., *Q. baishensis* (GenBank accession code: KP229805), and *Q. yalin* sp. nov. are 10.3% and 16.1% based on K2P, respectively.

### Qiongthela yalin sp. nov.

Taxon classificationAnimaliaAraneaeLiphistiidae

FA543615-329D-52DF-AC21-26B9D7877748

http://zoobank.org/FDD06B09-7B47-4055-84F4-4BD552242BD2

Figures 6 , 7

#### Type material.

***Holotype*** ♂: China, Hainan Province, Sanya City, Yalinling, 18.51°N, 109.24°E, alt. 220 m, 22 August 2019, D. Li, F.X. Liu, X. Xu and L. Yu leg., XUX–2019–140 (matured on 2 October 2019 at HNU). ***Paratypes***: 1 ♂, 2 ♀♀, same data as holotype; XUX–2019–138 (♂ matured on 6 November 2019 at HNU), XUX–2019–139, 141; 1 ♀; same locality as holotype, 18.50°N, 109.23°E, alt. 240 m, 1 August 2017, D. Li, F.X. Liu, Z.T. Zhang and X. Xu leg., XUX–2017–033.

#### Diagnosis.

Males of *Q. yalin* sp. nov. resemble those of *Q. sanya*, but can be distinguished from the latter by the narrower conductor base (Fig. 7A, D) and by the cymbium with a longer and more slender projection (Fig. 7G); from those of *Q. dongfang* sp. nov. by the tegular marginal apophysis slightly longer and with a blunt apex (Fig. 7A, D), and the cymbium with an elongated projection (Fig. 7G); from those of the rest of *Qiongthela* species by the contrategulum with a smooth edge proximally (Fig. 7F, G). Females of *Q. yalin* sp. nov. differ from those of *Q. sanya* by the middle receptacular clusters having short, indistinct genital stalks (Fig. 7I, L); from those of *Q. australis*, *Q. yini* and *Q. yinggezui* by the smaller middle receptacular clusters compared with the lateral ones (Fig. 7H–M); from those of the rest of *Qiongthela* species by the middle receptacular clusters situated along the anterior margin of the bursa copulatrix, the laterals located slightly on the dorsal wall of the bursa copulatrix, and the trapezoidal bursa copulatrix (Fig. 7H–M).

#### Description.

**Figure 6.**
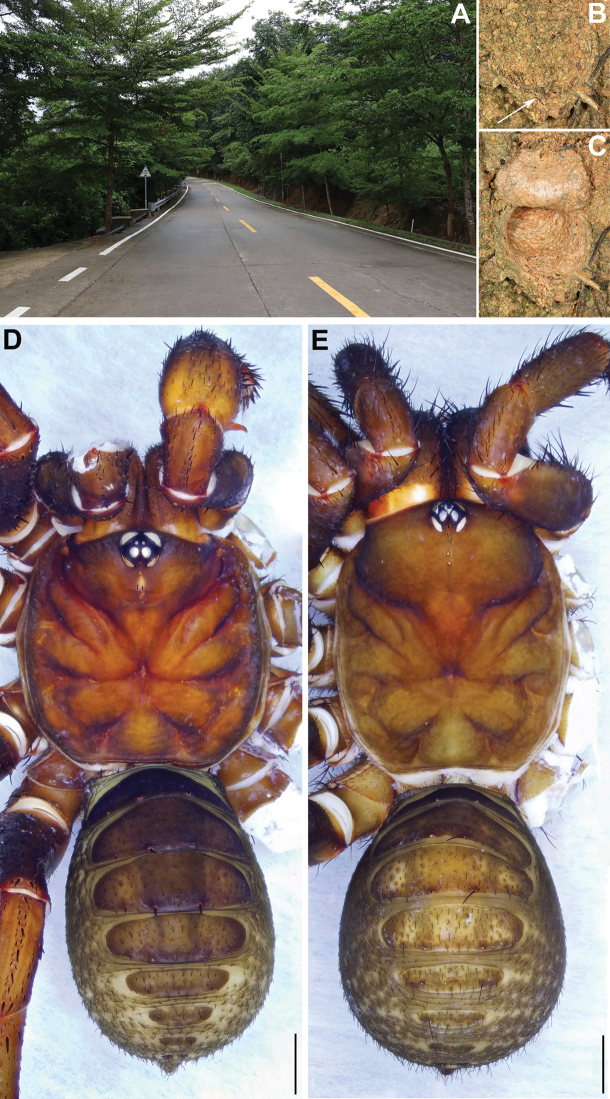
Microhabitat and general somatic morphology of *Qiongthela yalin* sp. nov. **A** microhabitat **B, C** trapdoor with door closed and open **D** male (XUX–2019–140, holotype) **E** female (XUX–2019–139); Scale bars: 2 mm (**D, E**).

**Male** (holotype, Fig. 6D). Carapace reddish brown; opisthosoma brown, with 12 dark brown tergites, close to each other, the first 2–7 larger than the others, and the fourth largest; sternum narrow, much longer than wide; a few fine pointed hairs running over the ocular area; chelicerae with promargin of cheliceral groove bearing 9 denticles of variable size; legs with firm hairs and spines; 7 spinnerets. Measurements: BL 16.80, CL 7.52, CW 7.72, OL 9.13, OW 6.52; ALE > PLE > PME > AME; leg I 26.07 (7.40 + 1.96 + 6.12 + 7.23 + 3.36), leg II 25.66 (7.00 + 1.92 + 5.81 + 7.21 + 3.72), leg III 25.51 (6.06 + 1.70 + 5.40 + 8.10 + 4.25), leg IV 33.43 (8.64 + 2.44 + 7.18 + 10.26 + 4.91).

**Figure 7.**
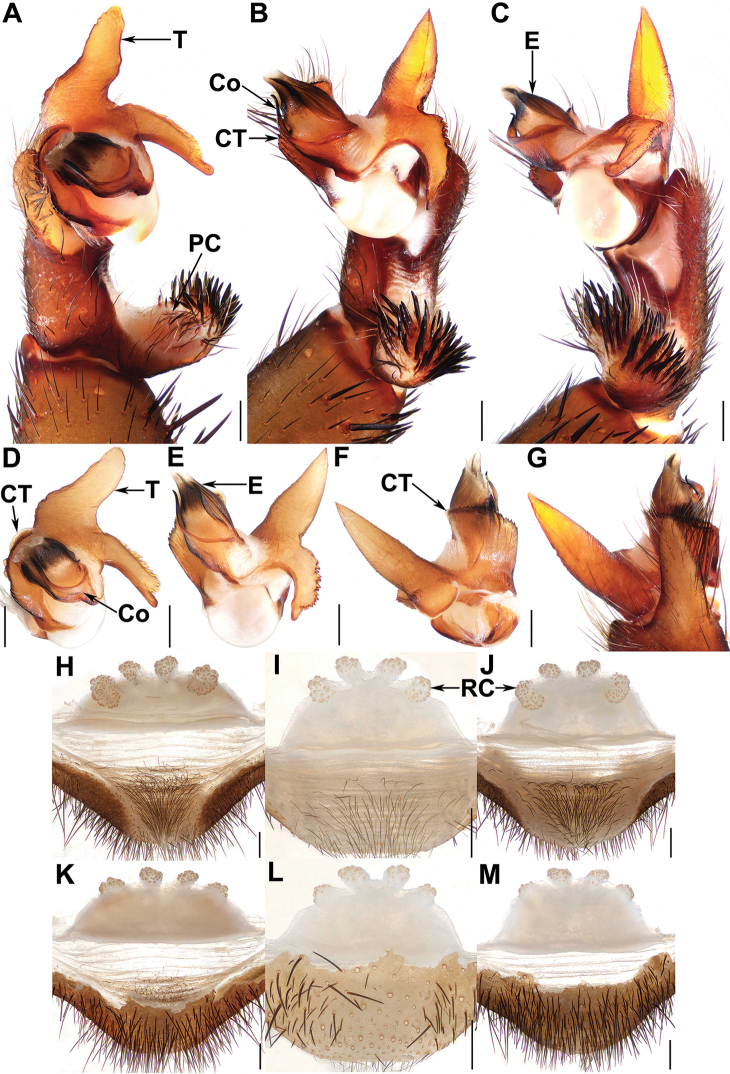
Male and female genital anatomy of *Qiongthela yalin* sp. nov. **A** left palp, prolateral view **B, E** left palp, ventral view **C** left palp, retrolateral view **D** left palp, distal view **F, G** left palp, dorsal view **H–J** vulva, dorsal view **K–M** vulva, ventral view **A–C, G** XUX–2019–140 (holotype) **D–F** XUX–2019–138 **H, K** XUX–2017–033 **I, L** XUX–2019–139 **J, M** XUX–2019–141; Scale bars: 0.5 mm.

***Palp*.** Cymbium with a slender projection dorsally (Fig. 7G); paracymbium unpigmented and unsclerotised prolaterally, with numerous setae at the tip (Fig. 7A–C). Contrategulum with a smooth edge proximally and two edges distally: the inner one with fine, small denticles, the outer one smooth, sharp, semi-translucent, fused with the inner at the middle of the contrategulum (Fig. 7A, D, F). Tegulum with a long, slightly curved, distally blunt marginal apophysis (Fig. 7A, D), the proximally directed terminal apophysis with a dentate margin, continuously narrowing to a rounded, hooked apex (Fig. 7A–E). Conductor situated ventro-proximally on the embolus, fused with the embolus at the basal portion, distal portion free, narrowing to a slightly bent apex (Fig. 7A–E). Embolus largely sclerotised, with a wide, flat opening of the sperm duct distally, ventrally smooth, retrolaterally with several longitudinal ribs (Fig. 7B, C, E).

**Female** (Fig. 6E). Carapace light brown; opisthosoma brown, with 12 brown tergites, separate from each other, the first 2–7 larger than the others, and the fourth largest; sternum narrow, nearly twice as long as wide; a few fine pointed hairs running over the ocular area; chelicerae robust with promargin of cheliceral groove containing 9 denticles of variable size; legs with firm hairs and spines; 7 spinnerets. Measurements: BL 18.31, CL 8.60, CW 7.47, OL 9.16, OW 7.52; ALE > PLE > PME > AME; palp 14.72 (5.12 + 1.88 + 3.53 + 4.19), leg I 17.23 (5.56 + 2.14 + 3.82 + 3.61 + 2.10), leg II 16.69 (5.46 + 2.22 + 3.42 + 3.33 + 2.26), leg III 17.17 (5.32 + 2.40 + 3.07 + 3.97 + 2.41), leg IV 24.44 (7.27 + 2.34 + 5.28 + 6.25 + 3.30).

***Female genitalia*.** The middle receptacular clusters along the anterior margin of the bursa copulatrix, the lateral ones located slightly on the dorsal wall of the bursa copulatrix; the middle ones smaller than the lateral ones, with indistinct genital stalks; bursa copulatrix trapezoidal (Fig. 7H–M).

#### Variation.

Males and females vary in body size. Range of measurements in males (*N* = 2): BL 15.76–16.80, CL 6.92–7.52, CW 6.18–7.72, OL 7.48–9.13, OW 5.89–6.52; in females (*N* = 3): BL 18.31–29.27, CL 8.60–14.19, CW 7.47–11.67, OL 9.16–13.68, OW 7.52–11.08.

#### Etymology.

The species epithet, a noun in apposition, refers to the type locality.

#### Distribution.

Hainan (Sanya), China

#### GenBank accession number.

XUX–2019–140: MT900753.

#### Remarks.

Both maximum and mean intraspecific genetic distances of *Q. yalin* sp. nov. are 0% based on K2P (*N* = 5). The interspecific genetic distance between *Q. yalin* sp. nov. and *Q. sanya* (GenBank accession code: MN911990) is 7.2% based on K2P.

## Supplementary Material

XML Treatment for Qiongthela

XML Treatment for Qiongthela dongfang

XML Treatment for Qiongthela nankai

XML Treatment for Qiongthela yalin
